# Plant-Based Diets and Their Associations with Physical Performance in the Baltimore Longitudinal Study of Aging

**DOI:** 10.3390/nu16234249

**Published:** 2024-12-09

**Authors:** Galya Bigman, Marius Emil Rusu, Amber S. Kleckner, John D. Sorkin, Yichen Jin, Sameera A. Talegawkar, Toshiko Tanaka, Luigi Ferrucci, Alice S. Ryan

**Affiliations:** 1 Division of Gerontology, Department of Epidemiology and Public Health, University of Maryland School of Medicine, Baltimore, MD 21201, USA; 2 Department of Pharmaceutical Technology and Biopharmaceutics, Faculty of Pharmacy, Iuliu Hatieganu University of Medicine and Pharmacy, 400012 Cluj-Napoca, Romania; rusu.marius@umfcluj.ro; 3 Department of Pain and Translational Symptom Science, University of Maryland School of Nursing, Baltimore, MD 21201, USA; amber.kleckner@umaryland.edu; 4 Baltimore Veterans Affairs Medical Center, Division of Gerontology, Geriatrics and Palliative Medicine, Department of Medicine, University of Maryland School of Medicine, Baltimore, MD 21201, USA; jsorkin@som.umaryland.edu (J.D.S.); aryan@som.umaryland.edu (A.S.R.); 5 Baltimore Geriatric Research, Education and Clinical Center, Veterans Affairs Maryland Health Care System, Baltimore, MD 21201, USA; 6 Department of Exercise and Nutrition Sciences, Milken Institute School of Public Health, The George Washington University, Washington, DC 20037, USA; yjin@gwu.edu (Y.J.); stalega1@email.gwu.edu (S.A.T.); 7 Longitudinal Studies Section, Translational Gerontology Branch, National Institute on Aging, Baltimore, MD 21224, USA; tanakato@mail.nih.gov (T.T.); ferruccilu@grc.nia.nih.gov (L.F.)

**Keywords:** physical function, short physical performance battery, grip strength, gait speed, older adults, nutrition, aging, muscle mass, muscle strength

## Abstract

Background: Plant-based diets are associated with various health benefits; however, their impact on physical performance in aging populations remains unclear. Objectives: To investigate the associations between adherence to plant-based diets and physical performance, focusing on their potential protective effects against age-related declines in function. Methods: Data were obtained from men and women aged 40 years or older in the Baltimore Longitudinal Study of Aging (BLSA) (mean ± SD age: 68 ± 13 years at the first dietary visit; n = 1389). Dietary intake was assessed using a food frequency questionnaire (FFQ). Plant-based diets, calculated from 18 food groups, were categorized as overall (PDI), healthful (hPDI), or unhealthful (uPDI), and their tertiles across visits were analyzed. Multivariable linear mixed-effects models were used to examine the association between repeated measurements of three physical performance outcomes—Short Physical Performance Battery (SPPB), grip strength (kg), and gait speed (m/s)—and adherence to each plant-based diet. Results: In fully adjusted models, SPPB and grip strength were significantly associated with both hPDI and uPDI, but not with PDI. For hPDI, the intermediate tertile showed the greatest benefit, with SPPB scores 0.5 points higher (β_T2vs.T1_ = 0.50, 95% CI: 0.30–0.70, *p* < 0.001) over the follow-up period. In contrast, for uPDI, a 0.27-point lower SPPB score was seen (β_T3vs.T1_ = −0.27, 95% CI: −0.48 to −0.07, *p* = 0.009). Longitudinally, grip strength was positively associated with hPDI (β_T3vs.T1_ = 1.14, 95% CI: 0.24–2.05, *p* = 0.0013). Similar results were observed in older adults aged ≥65 years. Conclusions: Adherence to hPDI may benefit lower body function and muscle strength, while uPDI appears to have adverse effects. This suggests that the quality of plant-based foods is essential for maintaining functional well-being in older adults. Further research is needed to confirm these findings, explore underlying mechanisms, and identify strategies to optimize plant-based dietary patterns for aging populations.

## 1. Introduction

Physical performance in older adults is fundamental to promoting healthy aging and minimizing the risk of morbidity and disability [1,2,3,4]. Research frequently assesses physical performance through measures such as handgrip strength, lower body strength, balance, and mobility, which are closely tied to skeletal muscle mass, strength, and function [1,2,3,4,5]. Age-related declines in muscle mass and strength, which typically begin in adulthood and accelerate with age, are well documented, with muscle mass declining by approximately 1–3% per year and muscle strength by 2.5–4% per year [5]. Factors such as disuse and malnutrition can exacerbate these declines, contributing to impaired physical performance in aging individuals [3].

Dietary intake plays a significant role in supporting muscle health and mitigating age-related declines in physical performance [1,6]. Protein, in particular, is essential for preserving muscle mass and function throughout the lifespan [7,8]. Emerging research has also explored the effects of dietary patterns and specific nutrient-dense foods, especially phytonutrient-rich fruits and vegetables, on muscle health [9,10,11,12]. Such foods have been associated with benefits including improved grip strength [11,12] and physical function (13,14), likely due to their antioxidative and anti-inflammatory compounds (e.g., carotenoids, polyphenols, and flavonoids) [11,12,13,14].

Dietary patterns emphasizing plant-based foods, such as the DASH diet [15], the Nordic diet [16], and the Mediterranean diet, have been associated with improved physical function and strength in older adults [15,17,18,19]. Findings from the Baltimore Longitudinal Study of Aging (BLSA) showed that, over a median follow-up period of six years, participants in the highest tertile of adherence to the MIND diet—a variation of the Mediterranean and DASH diets—had 57% lower odds of functional impairment, experienced slower functional decline, and demonstrated greater grip strength compared to those in the lowest tertile [15]. Similarly, adherence to the Mediterranean diet was positively associated with fat-free mass and leg explosive power, though not with grip strength among women [18], except in active elderly women from Italy and the Netherlands [17]. These dietary patterns consist of a mix of plant-based foods alongside moderate amounts of fish, poultry, dairy, and limited red meat [11,12,13,14,15,16,17,18].

Despite the established benefits of these well-studied dietary patterns, there is a growing shift across all age groups toward higher adherence to plant-based eating patterns, driven by concerns over health, sustainability, cultural norms and traditional foodways, and animal welfare [20,21]. These diets vary widely in composition, encompassing both nutrient-dense foods (e.g., whole grains, vegetables, and fruits) and less nutritious options (e.g., refined grains, sugary beverages, and sweets) [21]. While the health benefits of plant-based diets for conditions such as cardiovascular disease [22,23], diabetes [24], and cancer [22,23] are well documented [25,26], their effects on physical function and performance, particularly in aging populations, are less studied and remain inconclusive [27,28,29].

In plant-based diets, replacing animal products with plant-based alternatives even partly may impact muscle health in the long term; such alternatives may not fully meet the specific nutrient requirements essential for muscle health and function. A systematic review of 17 studies examining the effects of a plant-based diet, which includes fewer animal products, on physical performance and body composition in middle-aged and older adults found mixed results. Two of three studies found positive associations between plant-based diets and physical performance, though some measures, like gait speed, showed no significant link [27].

The limited number of studies, coupled with mixed findings and the potential for greater adherence to plant-based diets, underscores the need for rigorous, longitudinal research to elucidate the risks of such diets on muscle health and function. This study aimed to address these gaps by investigating the long-term longitudinal relationships between plant-based diets (distinguishing between healthy and unhealthy versions) and physical performance, including muscle strength, mobility, and function, in an aging population. Using data from the BLSA [30], we hypothesize that adherence to a healthy plant-based diet is positively associated with physical performance, while an unhealthy plant-based diet is linked to poorer performance. This work contributes to a more nuanced understanding of dietary impacts on aging, helping to inform dietary recommendations for older adults.

## 2. Materials and Methods

### 2.1. Study Population

The data were derived from the BLSA, an ongoing, open cohort study initiated in 1958 and currently conducted by the Intramural Research Program of the National Institute on Aging (IRP-NIA). Comprehensive descriptions of the BLSA cohort and study protocols have been previously published [13,15,30].

In brief, individuals who were living independently in the Baltimore–Washington area in the United States were recruited into the study. Follow-up assessments were conducted every four years for participants younger than 60 years, every two years for those aged 60 to 79 years, and annually for those aged 80 years and above. During these assessments, interviews, clinical examinations, and laboratory tests were performed by the study staff, either at the IRP-NIA Clinic Research Unit or, in cases of severe debilitation, through home visits. In 2005, the BLSA started collecting dietary data using a food frequency questionnaire (FFQ), which has been validated against diet records [31].

For the purposes of this study, data collected between 2005 and 2018 were analyzed, including dietary data from 2005 to 2015 and physical function data from 2005 to 2018. The study protocol was reviewed and approved by the Institutional Review Board (IRB) of the National Institute of Environmental Health Sciences, and participants provided informed consent at each visit.

**Figure 1:**
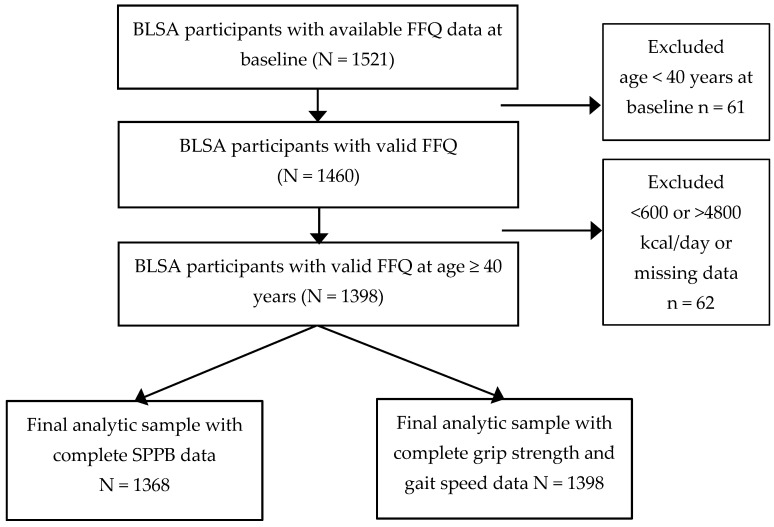
Participant flowchart at baseline. BLSA, Baltimore Longitudinal Study of Aging. SPPB, Short Physical Performance Battery.

A total of 1521 middle-aged or older participants with valid FFQ data were included. Exclusions included those with energy intake <600 or >4800 kcal/day or missing data (n = 62), age < 40 years (n = 61), and missing physical function data (n = 30), Figure 1.

### 2.2. Dietary Intake Assessment

Self-reported dietary intake over the past year was collected during each visit using a semi-quantitative FFQ consisting of intake for 223 food line-items [31]. This FFQ was designed for use in the general U.S. population and was self-administered. Upon completion, all FFQs were reviewed for completeness prior to being scanned and processed at the Human Nutrition Research Center on Aging at Tufts University in Boston, MA. Nutrient and energy intake estimates were generated using the Nutrient Data System for Research (NDSR) software developed by the University of Minnesota [32].

#### Plant-Based Diet Indices

Adherence to a plant-based diet was assessed by the overall plant-based diet index (PDI), the healthful plant-based diet index (hPDI), and the unhealthful plant-based diet index (uPDI) [26]. These indices encompass a total of 18 food groups, categorized as follows: seven are designated as healthy plant-based groups (whole grains, fruits, vegetables, nuts, legumes, vegetable oils, and tea and coffee), five as unhealthy plant-based groups (fruit juices, refined grains, potatoes, sugar-sweetened beverages, and sweets), and six as animal-based food groups (animal fats, dairy products, fish, meat, eggs, and miscellaneous animal-based foods) (Supplementary Table S1).

Dietary indices were scored by stratifying food group intake levels (in grams) into cohort-specific energy-adjusted quintiles, assigning a score of 1 to 5 to each quintile. In the overall plant-based diet index (PDI), both healthful and unhealthful plant-based food groups were assigned positive scores, indicating that higher consumption levels correlated with higher scores. In the healthful plant-based diet index (hPDI), healthful plant food groups were awarded positive scores, while unhealthful plant foods received inverse scores. Conversely, in the unhealthful plant-based diet index (uPDI), healthful plant foods were assigned inverse scores, and unhealthful plant foods received positive scores. Across all three indices, animal-based food groups were scored inversely. The scores for the 18 food groups categorized by quintiles were aggregated to derive the final index scores [26].

The final PDI, hPDI, and uPDI were further stratified into tertiles (T1, T2, and T3) based on the distribution of participants’ scores, with T3 indicating higher adherence to the examined diet and T1 serving as the reference in the multivariable models.

### 2.3. Physical Performance

Physical function was assessed using the Short Physical Performance Battery (SPPB) during each follow-up visit, as described by previous studies [13,15,33]. The SPPB evaluates lower body performance through three tests: (1) repeated chair stands, which measures the time taken to rise from a chair five times; (2) progressive standing balance, which assesses the ability to maintain side-by-side, semi-tandem, and full-tandem stances for 10 s each; and (3) usual gait speed, measured over a 6 m distance. Each test is scored from 0 to 4, with a score of 4 indicating the best performance. The scores from the three tests are summed to generate an overall SPPB score, ranging from 0 to 12 [33].

Grip strength was assessed at each follow-up visit using a Smedley Hand Dynamometer (Stoelting). The dynamometer was calibrated and adjusted to fit each participant’s hand grip to ensure accuracy, after which the participant would squeeze as hard as they could. Measurements were taken for both hands, and the average of these measurements, in kilograms (kg), was used for subsequent analysis [11,15].

Gait speed was measured over a fixed 6 m distance. Participants walked at their usual pace, and the time taken to cover the distance was recorded. Gait speed (meters per second) was calculated by dividing the distance by the time. Participants were allowed to use mobility aids if needed.

Covariates were selected based on prior research and included age, sex, race/ethnicity, years of education, smoking status, body mass index (BMI), physical activity, number of chronic diseases at the time of the first diet visit, time since the first diet visit, alcohol intake in the past 12 month in gram/day, and average energy intake across all valid diet assessments [13,15]. With the exception of BMI, all covariates were evaluated through a structured questionnaire. Race/ethnicity was self-reported and categorized into non-Hispanic White, non-Hispanic Black/African American, or other. Smoking status was classified into three categories: never smoker, former smoker, or current smoker. Weight and height were measured without shoes using a Detecto medical beam scale, and BMI (kg/m^2^) was calculated as weight (kg) divided by the square of height (m). Physical activity was assessed using a validated leisure-time physical activity questionnaire [34], which captured the duration and frequency of various sports and recreational activities. Based on Metabolic Equivalent of Task (MET) minutes per week, participants were categorized into four activity levels: sedentary (<50 MET-min/week), low (50–249 MET-min/week), moderate (250–499 MET-min/week), and high (≥500 MET-min/week). The number of chronic diseases, including anemia, cancer, congestive heart failure, cognitive impairment, chronic kidney disease, chronic obstructive pulmonary disease, depressive symptoms, diabetes, hip replacement, heart disease, hypertension, joint pain, peripheral artery disease, Parkinson’s disease, and stroke, was determined using the International Classification of Diseases, Ninth Revision (ICD-9) codes, and/or through self-report [35]. Although alcohol intake (g/day) was not included in the indices, consumption was included in the analysis as a covariate. Energy intake was estimated from the food frequency questionnaire (FFQ) at each visit and averaged across all available visits.

### 2.4. Statistical Analysis

Differences in participant characteristics among the tertiles of the plant-based diet indices (PDI, hPDI, and uPDI) were analyzed using analysis of covariance (ANOVA) for continuous variables where the Bonferroni correction was applied to adjust for multiple comparisons and the chi-square test for categorical variables. Data are presented as n (%) for categorical variables and means ± standard deviations (SDs) for continuous variables.

We employed mixed-effects modeling to account for both fixed and random effects when investigating the association between adherence to plant-based diet indices (PDI, hPDI, uPDI, and tertiles) and three outcomes: physical function, grip strength, and gait speed. A random intercept and slope for time since the first diet visit were included to capture individual variability over time adjusting for age, sex, race, and education (Model-1). Fully adjusted models accounted for additional covariates, including smoking status, physical activity, alcohol intake, number of chronic diseases, BMI, years since the first diet visit, and average energy intake (Model-2).

Multiple covariance structures were evaluated to determine the best fit for the data, including independent, exchangeable, and unstructured covariance matrices. Model selection was based on the Akaike Information Criterion (AIC) and Bayesian Information Criterion (BIC), where lower values indicate a better balance between model fit and complexity. We compared several models and found that the model with an unstructured covariance structure had the lowest AIC and BIC, indicating the best fit. Therefore, the unstructured covariance model was selected as the final model for all our analyses.

Lastly, in separate models, we restricted the analysis among adults aged 65 years and older at baseline using the mixed-effects models to examine the association between adherence to hPDI and the three physical performance outcomes. For presentation and comparison purposes, we standardized physical performance outcomes, allowing their final coefficients to be displayed on the same graph.

Participants with missing data for any variable were excluded from the regression analyses (n = 62). All statistical analyses were conducted using STATA version 18.0 (StataCorp LLC, College Station, TX, USA), with a two-tailed type I error rate (α = 0.05) used to determine statistical significance.

## 3. Results

### 3.1. Baseline Characteristics by Plant-Based Diet Indices

Table 1 presents participant characteristics and their distribution across plant-based diet indices (PDI, hPDI, and uPDI). The mean baseline age was 68.3 ± 12.8 years; 46.8% were male, and 72.3% were Non-Hispanic White. Overall, 38.7% were overweight and 23.4% were obese. Characteristics varied by PDI tertiles, with more women in the top tertiles of both PDI (58.7%) and hPDI (61.6%). Older age correlated with higher PDI (*p* = 0.018) and hPDI (*p* = 0.001) but not uPDI (*p* = 0.30).

Higher PDI tertiles were associated with progressively greater intake of whole grains (1.65 to 2.08; *p* < 0.001), fruits (0.98 to 1.68; *p* < 0.001), vegetables (2.53 to 3.20; *p* < 0.001), nuts (0.55 to 0.87; *p* < 0.001), beans (0.14 to 0.25; *p* < 0.001), and vegetable oils (0.18 to 0.35; *p* < 0.001). Similarly, higher PDI tertiles also corresponded to increased intake of unhealthy foods such as juices (0.42 to 0.65; *p* < 0.001), sugar-sweetened beverages (0.73 to 0.91; *p* < 0.001), and sweets (0.88 to 0.99; *p* < 0.001) (Supplementary Table S2).

**Figure 2:**
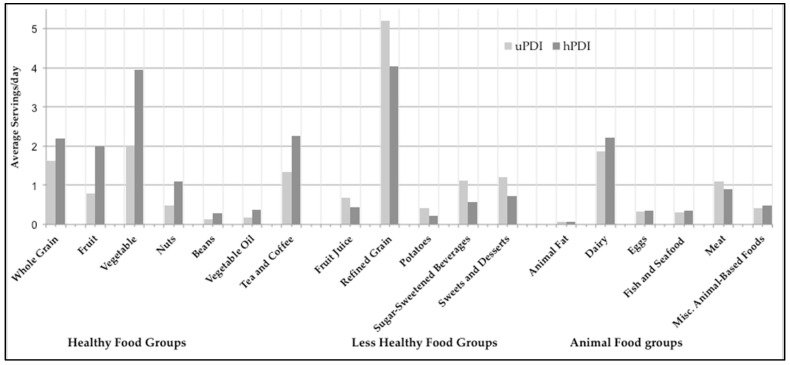
Comparison of average daily servings for each food group between the top tertiles of the healthy (hPDI, dark gray) and unhealthy (uPDI, light gray) dietary indices. Misc: miscellaneous.

Participants in the highest tertile of the hPDI had significantly greater intakes of all healthy food groups and lower intakes of all less healthy food groups compared to those in the highest tertile of the uPDI (*p* < 0.001). No differences were observed between hPDI and uPDI in the intakes of eggs, fish and seafood, and animal fat (*p* > 0.2) (Figure 2).

### 3.2. Physical Performance and Plant-Based Diet Indices

ANOVA was used to assess differences in SPPB scores across tertiles of the three plant-based indices (Table 2). Across PDI tertiles, gait speed and SPPB did not vary. However, participants in the highest tertile of hPDI had significantly higher SPPB scores (*p* < 0.001) and higher gait speed, while those in the highest tertile of uPDI had significantly lower SPPB scores (*p* < 0.001). For grip strength, we observed a different trend: participants in the highest tertile of PDI had significantly lower grip strength (*p* < 0.001), consistent with the relationship between uPDI score and grip strength (*p* < 0.001), though higher hPDI was not associated with lower grip strength (*p* = 0.436).

In multivariable analysis creating adjusted models (Model-1 and Model-2), no significant associations were found between overall PDI and physical performance. Therefore, when PDI was separated into hPDI and uPDI, distinct patterns emerged in relation to physical performance outcomes. Specifically, hPDI was positively associated with measures of physical performance, while uPDI showed negative associations (Table 3). For example, hPDI was associated with higher SPPB scores, with tertile 3 scoring 0.25 points higher than tertile 1 (95% CI: 0.05–0.46, *p* = 0.017) and tertile 2 scoring 0.50 points higher than tertile 1 (95% CI: 0.30–0.70, *p* < 0.001).

Conversely, participants in the highest uPDI tertile (tertile 3) had a 0.27-point lower SPPB score compared to those in tertile 1 (β = −0.27 [95% CI: −0.48 to −0.07], *p* = 0.009). Grip strength was positively associated with hPDI (β = 1.14 [95% CI: 0.24 to 2.05], *p* = 0.013) and negatively associated with uPDI in Model-1 (β = −1.05 [95% CI: −1.98 to −0.13], *p* = 0.084). 

### 3.3. Physical Performance and Plant-Based Diet Indices in Older Adults

**Figure 3:**
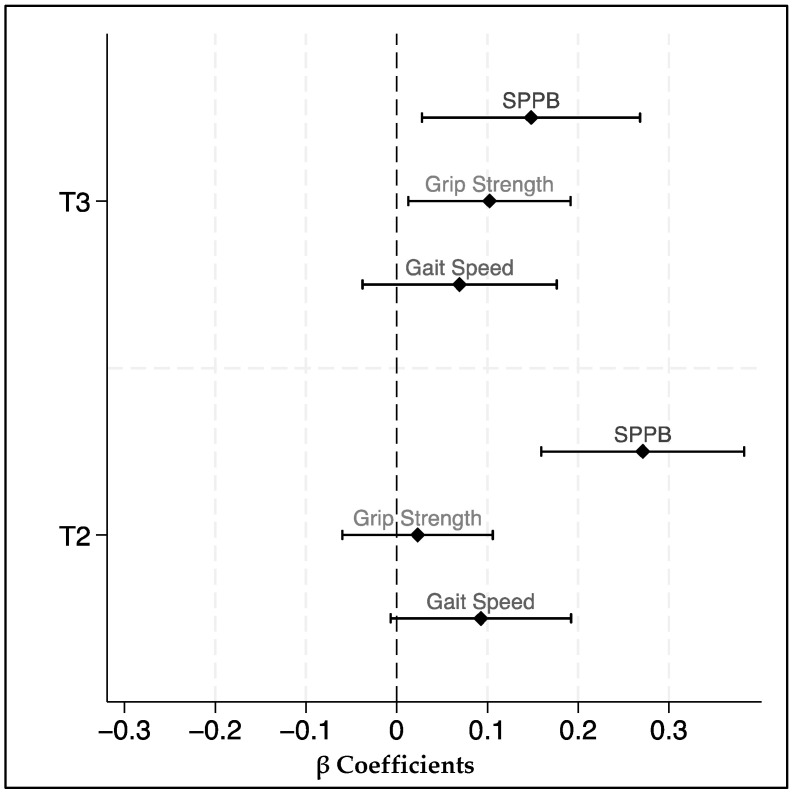
Associations between healthy plant-based diet adherence (hPDI) by tertiles (T3 and T2 vs. T1) and standardized physical performance outcomes in older adults aged 65 years or older. T3: higher adherence; T1: reference group.

A sensitivity analysis was conducted in a subset of participants aged 65 and older (n = 845, 60.8% of the sample). The results were consistent the prior analysis where higher adherence to a healthy plant-based diet was significantly associated with greater SPPB and grip strength. No significant associations were observed for PDI. The outcomes were standardized, allowing for a comparative evaluation of their beta coefficients, as illustrated in Figure 3.

## 4. Discussion

This research provides timely insights into the implications of adherence to plant-based diets on muscle health and function in aging populations. Given the growing popularity of these diets due to potential health benefits and environmental and ethical considerations [20,21], understanding their long-term effects on muscle health and function becomes increasingly important, especially as populations age.

Our findings, based on U.S. middle-aged and older adults from the BLSA cohort followed over 12 years, reveal that general adherence to a plant-based diet is not associated with improved physical performance, with no observed benefits on muscle health even after comprehensive adjustments. However, adherence to a healthy plant-based diet—emphasizing nutrient-dense food groups such as fruits, vegetables, whole grains, nuts, and beans—shows a positive association with physical function and strength, suggesting potential benefits for maintaining muscle strength and functional capacity.

Similarly, in our study, findings among adults aged 65 and older showed that higher adherence to a healthy plant-based diet was associated with better physical function and grip strength, highlighting its potential to support resilience in later life. This association remained robust even after adjusting for socio-demographics, lifestyle factors, and comorbidity load.

Conversely, a plant-based diet characterized by a high intake of unhealthy foods (uPDI)—such as refined grains, sugar-sweetened beverages, and juice—was negatively associated with physical function, may not support muscle function, and could even contribute to negative health effects. This highlights the importance of focusing on the quality of plant-based foods within the diet for optimal muscle health and function

In our study, the intake of animal-sourced foods was relatively similar between the healthy and unhealthy plant-based diet indices (PDIs) groups; key distinctions instead lay in the higher intake of fruits, vegetables, whole grains, nuts, and coffee/tea. These nutrient-dense food groups along with the less healthy foods likely contributed to the observed differences in physical function and grip strength. The findings suggest that the diet quality of plants may play a more pivotal role on physical performance than simply including or excluding meat or animal products in supporting physical performance.

In our analysis, SPPB and gait speed demonstrated better functional outcomes at the intermediate tertile of the hPDI, whereas grip strength was optimal in the highest tertile. This variation might reflect the unique demands of SPPB and gait speed, both of which are dynamic measures influenced by neuromuscular coordination and cognitive function, factors that may respond differently to components of a healthy plant-based diet. In a similar study [15], the authors found an inverse association between the MIND diet scores—emphasizing phytonutrient-rich foods and fish for omega-3s, crucial for cognitive health—and physical function impairment (per 1-point increment). These findings suggest that the trend toward reducing or replacing animal products with plant-based alternatives, without prioritizing nutrient quality, could have long-term implications for muscle health, as essential nutrients for muscle function may be lacking in diets that are plant-based but nutritionally imbalanced [27,36].

The findings on grip strength in this study are consistent with our previous study using the Healthy Eating Index-2015 (HEI-2015) within the National Health and Nutrition Examination Survey (NHANES), which found that higher diet quality—marked by sufficient intake of protein as well as whole grains, greens and beans, vegetables, and whole fruits—was associated with a reduced risk of low grip strength compared to lower-quality diets [11].

Few studies have explicitly examined adherence to plant-based diets and their influence on muscle health and function [27]. One study from the Chinese Longitudinal Health Longevity Survey found that a plant-based diet reduced the risk of muscle mass loss in functionally independent older adults, while a combination of plant-based and animal-based foods was more effective for those with functional impairments [37]. A longitudinal study conducted in England, Wales, and Scotland, using data from the National Survey of Health and Development with 969 participants, assessed dietary patterns through principal component analysis. While it did not specifically examine hPDI, the study identified a high-quality diet characterized by greater consumption of fresh fruit, leafy vegetables, and wholegrain bread, and lower consumption of white bread, added sugar, and processed meat. The findings highlighted that maintaining such a diet throughout adulthood is associated with improved physical performance in older age, including faster chair rise speed and longer standing balance time [38].

Healthful plant-based diets may protect against declines in physical function and muscle health by mitigating frailty. In a cohort of 24,996 individuals aged 40–70 years, greater adherence to hPDI was associated with a lower risk of frailty, while adherence to uPDI increased frailty risk after a median follow-up of 6.7 years [39]. Similarly, in the Nurses’ Health Study of 82,234 women aged ≥ 60 years, a 10-unit increment in hPDI was associated with a 15% lower risk of frailty, measured using the RAIL scale (fatigue, low strength, reduced aerobic capacity, multiple illnesses, and significant weight loss) [40]. A systematic review of 12 studies involving 20,518 participants found that higher adherence to a Mediterranean diet was inversely associated with frailty (OR 0.42, 95% CI: 0.28–0.65) and functional disability (OR 0.75, 95% CI: 0.61–0.93) in community-dwelling older adults aged 60 and over [41]. Notably, adherence to a Mediterranean diet, which prioritizes plant-based components such as fruits, vegetables, and whole grains, has been linked to reduced inflammatory markers, improved physical performance, and decreased frailty in older adults [41,42,43,44].

A healthy plant-based diet also provides several essential nutritional factors beneficial for muscle health and age-related muscle preservation, beyond the well-established role of protein and specifically branched-chain amino acids (BCAAs), which are primarily derived from animal sources [45]. Key nutrients include essential amino acids from plants, as well as vitamins D and K, carotenoids, vitamins C and E, minerals, dietary fats (notably n-3 fatty acids), polyphenols, prebiotics, and fibers. These components may enhance muscle mass, strength, and body composition in sarcopenic patients by modulating anti-inflammatory, antioxidant, and anabolic processes [46,47,48,49,50].

Dietary patterns high in exogenous antioxidants such as the hPDI can mitigate oxidative stress, reducing cellular senescence and maintaining redox balance in muscle cells. Additionally, bioactive compounds in these diets may lower chronic inflammation by modulating signaling pathways like nuclear factor κB (NF-κB), which controls pro-inflammatory cytokine expression (e.g., TNF-α, IL-6, and IL-8) [50,51]. Another benefit of plant-based diets is the production of short-chain fatty acids (SCFAs) by gut microbiota from plant fibers. SCFAs support muscle health by enhancing insulin sensitivity and maintaining muscle homeostasis via the gut–muscle axis. Increased SCFA synthesis can activate AMPK, a protein expressed in various tissues, which stimulates skeletal muscle fatty acid oxidation and glucose uptake. Additionally, it activates PGC-1α, a key regulator of mitochondrial biogenesis and cellular energy management [52]. Moreover, SCFAs, particularly butyrate, have anti-sarcopenic effects, contributing to muscle protein synthesis and mitigating muscle wasting [52,53,54,55].

However, the results should be interpreted with caution as this study has certain limitations. The BLSA cohort primarily consists of individuals with relatively high physical performance and higher education levels, potentially limiting the generalizability of findings to the broader U.S. aging population. Dietary intake was assessed using a validated food frequency questionnaire (FFQ), which, while effective for capturing long-term dietary patterns, has inherent limitations in estimating absolute nutrient values and precise intake levels. These limitations were partially mitigated by focusing on overall dietary patterns rather than specific nutrient quantities and by using cumulative dietary data across assessments to reduce potential misclassification. Furthermore, as an observational study, our findings do not imply causality, and despite comprehensive adjustments, the possibility of residual confounding remains. Although we adjusted for overall calorie intake in our model, we did not adjust for protein intake, which is crucial for muscle health and function and may vary across diets.

Nonetheless, this study’s strengths include addressing a gap in the field, as current research in this area is limited. Dietary intake was averaged across multiple visits, which improves the precision of the estimates of dietary intake. We also evaluated physical function using several different measures, providing a comprehensive assessment of physical function. Its long-term follow-up, using a longitudinal design with repeated measurements of physical performance and comprehensive adjustments for sociodemographic and lifestyle factors, enhances the robustness of our findings on plant-based diets and physical function in aging.

## 5. Conclusions

In conclusion, while general adherence to a plant-based diet did not show significant improvements in physical performance, our findings indicate that a high-quality, healthy plant-based diet—high in nutrient-dense foods such as fruits, vegetables, whole grains, nuts, and beans—can positively influence physical function and grip strength in middle-aged and older adults. This highlights the importance of dietary quality within plant-based eating patterns for supporting muscle health and functional capacity, especially as individuals age. Conversely, plant-based diets high in refined grains, sugary beverages, and processed foods may be less effective or even detrimental for physical function, underscoring the need to focus on nutrient-rich, whole food choices within plant-based diets. These results emphasize the importance of distinguishing between healthy and unhealthy plant-based diets when evaluating their effects on physical performance in aging populations. Further research, both longitudinal and interventional, is needed to confirm these findings, uncover mechanisms, and refine dietary recommendations for optimizing plant-based diets to support physical performance in aging populations.

## Figures and Tables

**Table 1:** Baseline characteristics of study participants by plant-based diet indices (PDI, hPDI, and uPDI). N = 1389.

First Diet Visit	Overall	PDI				hPDI				uPDI			
N (%)/Mean ± SD		N (%)/Mean ± SD			*p*-Value	N (%)/Mean ± SD			*p*-Value	N (%)/Mean ± SD			*p*-Value
Characteristics		T1	T2	T3		T1	T2	T3		T1	T2	T3	
Sample Size	1389 (100)	471 (34)	501 (36)	417 (30)		487 (35.0)	466 (33.6)	436 (31.4)		469 (33.8)	460 (33.1)	460 (33.1)	
Age (years)	68.3 ± 12.8	66.9 ± 12.4	69.0 ± 12.8	68.9 ± 12.9	0.018	69.8 ± 13.1	68.0 ± 12.6	66.8 ± 12.4	0.001	67.6 ± 11.6	68.9 ± 12.5	68.3 ± 14.1	0.295
Race					<0.001				<0.001				0.034
NH-Black	313 (22.8)	90 (23.1)	120 (24.2)	103 (25.0)		129 (26.9)	104 (22.6)	80 (18.5)		87 (18.7)	115 (25.1)	111 (24.7)	
NH-White	992 (72.3)	361 (71.3)	359 (72.4)	272 (66.0)		341 (71.0)	341 (74.0)	310 (71.8)		347 (74.6)	322 (70.3)	323 (71.8)	
Other	68 (4.9)	14 (5.6)	17 (3.4)	37 (9.0)		10 (2.1)	16 (3.4)	42 (9.7)		31 (6.7)	21 (4.6)	16 (3.6)	
Sex					<0.001				<0.001				<0.001
Female	730 (53.2)	211 (45.4)	277 (55.9)	242 (58.7)		219 (45.6)	245 (53.2)	266 (61.6)		309 (66.5)	244 (53.3)	177 (39.3)	
Male	643 (46.8)	254 (54.6)	219 (44.2)	170 (41.3)		261 (54.4)	216 (46.8)	166 (38.4)		156 (33.5)	214 (46.7)	273 (60.7)	
BMI (kg/m^2^)					<0.001				<0.001				0.762
<25	525 (37.9)	147 (31.3)	193 (38.7)	185 (44.5)		147 (30.4)	187 (40.1)	191 (43.9)		185 (39.5)	177 (38.5)	163 (35.8)	
25–29	535 (38.7)	186 (39.7)	190 (38.1)	159 (38.2)		189 (39.1)	183 (39.3)	163 (37.5)		180 (38.5)	173 (37.6)	182 (39.9)	
≥30+	324 (23.4)	136 (29.0)	116 (23.3)	72 (17.3)		147 (30.4)	96 (20.6)	81 (18.6)		103 (22.0)	110 (23.9)	111 (24.3)	
Kcal/day	1995 ± 715	2161 ± 750	1915 ± 680	1904 ± 683	<0.001	1888 ± 700	1987 ± 728	2124 ± 699	<0.001	2018 ± 649	1862 ± 681	2104 ± 789	<0.001
Physical activity MET (minutes/day)					0.989				<0.001				0.001
<50	135 (9.8)	44 (9.5)	48 (9.6)	43 (10.3)		72 (14.9)	33 (7.2)	30 (6.9)		32 (6.9)	45 (9.9)	58 (12.7)	
50–250	538 (39.0)	182 (39.3)	199 (39.8)	157 (37.7)		205 (42.4)	184 (40.0)	149 (34.3)		159 (34.1)	189 (41.3)	190 (41.9)	
250–500	380 (27.6)	124 (26.7)	138 (27.6)	118 (28.3)		116 (24.0)	122 (26.5)	142 (32.7)		152 (32.5)	122 (26.7)	106 (23.4)	
>500	325 (23.6)	113 (24.4)	114 (22.8)	98 (23.5)		91 (18.8)	121 (26.3)	113 (26.0)		124 (26.5)	101 (22.1)	100 (22.0)	
Smoking history					0.037				0.529				0.017
Never smoked	867 (63.2)	289 (62.2)	298 (60.1)	280 (68.1)		303 (63.1)	284 (61.5)	280 (65.1)		273 (58.7)	309 (67.8)	285 (63.2)	
Former/Current smoker	505 (36.8)	176 (37.8)	198 (39.9)	131 (31.9)		177 (36.9)	178 (38.5)	150 (34.9		192 (41.3)	147 (32.2)	166 (36.8)	
Alcohol intake (gr/day)	13.0 ± 27.2	18.5 ± 33.4	12.2 ± 26.8	7.7 ± 16.5	<0.001	7.8 ± 13.8	12.3 ± 22.4	19.4 ± 39.2	<0.001	14.1 ± 25.7	10.9 ± 26.4	14.0 ± 29.5	0.144
Education (years)	17.6 ± 3.6	17.7 ± 5.0	17.5 ± 2.7	17.6 ± 2.7	0.723	17.3 ± 4.3	17.6 ± 3.9	18.0 ± 2.5	0.031	17.9 ± 2.4	17.6 ± 3.9	17.4 ± 4.4	0.091
Number of Chronic Diseases	2.35 ± 1.8	2.23 ± 1.83	2.39 ± 1.78	2.45 ± 1.68	0.289	2.54 ± 1.87	2.24 ± 1.76	2.25 ± 1.63	0.054	2.32 ± 1.71	2.33 ± 1.76	2.41 ± 1.84	0.827

T: Tertile; PDI: Plant-Based Diet Index; hPDI: Healthy PDI; uPDI: Unhealthy PDI; BMI: Body Mass Index; MET: Metabolic Equivalent of Task; NH: Non-Hispanic.

**Table 2:** Mean physical performance measures (SPPB, grip strength, and gait speed) by tertiles of plant-based diet indices (PDI, hPDI, and uPDI).

	Overall Plant-Based Diet Score	SPPB	Grip Strength (kg)	Gait Speed (m/s)
			Mean ± SD/(Range)	
Range		(0–12)	(3–182)	(0–2)
Overall Mean score		11.2 ± 2.0	30.6 ± 12.2	1.14 ± 0.28
PDI (30–75)				
T1	47.2 ± 3.6	11.3 ± 1.8	32.6 ± 14.5	1.15 ± 0.29
T2	54.5 ± 1.7	11.1 ± 2.1	29.5 ± 10.4	1.13 ± 0.28
T3	61.6 ± 3.4	11.1 ± 2.0	29.6 ± 11.3	1.12 ± 0.27
*p*-value	<0.001	0.216	<0.001	0.275
hPDI (33–80)				
T1	46.0 ± 3.5	10.7 ± 2.6	30.9 ± 13.1	1.09 ± 0.31
T2	53.9 ± 2.0	11.5 ± 1.3	30.8 ± 12.7	1.16 ± 0.24
T3	63.1 ± 4.7	11.3 ± 1.8	30.0 ± 10.6	1.16 ± 0.27
*p*-value	<0.001	<0.001	0.436	<0.001
uPDI (30–76)				
T1	44.9 ± 4.5	11.3 ± 1.6	29.0 ± 12.1	1.15 ± 0.25
T2	54.2 ± 2.0	11.2 ± 1.9	30.4 ± 10.4	1.14 ± 0.28
T3	62.6 ± 3.9	10.9 ± 2.4	32.4 ± 13.7	1.12 ± 0.30
*p*-value	<0.001	0.018	<0.001	0.245

T: Tertile; PDI: Plant-Based Diet Index; hPDI: Healthy PDI; uPDI: Unhealthy PDI; SPPB: Short Physical Performance Battery.

**Table 3:** Adjusted associations between plant-based diet indices (PDI, hPDI, and uPDI) and physical performance outcomes (SPPB, grip strength, and gait speed).

	β-Coefficient (CI 95%)								
	SPPB			Grip Strength (Kg)			Gait Speed (m/s)		
	Model-1	Model-2	*p*-Value *	Model-1	Model-2	*p*-Value *	Model-1	Model-2	*p*-Value *
PDI									
T1	ref	ref		ref	ref		ref	ref	
T2	0.20 (−0.03–0.43)	0.15 (−0.05–0.35)	0.139	0.17 (−0.72–1.06)	0.17 (−0.69–1.04)	0.696	0.02 (−0.00–0.05)	0.01 (−0.02–0.04)	0.213
T3	0.09 (−0.14–0.32)	0.07 (−0.13–0.27)	0.502	0.61 (−0.28–1.51)	0.65 (−0.22–1.54)	0.146	0.01 (−0.02–0.04)	0.01 (−0.02–0.03)	0.772
hPDI									
T1	ref	ref		ref	ref		ref	ref	
T2	0.69 (0.47–0.92)	0.50 (0.30–0.70)	<0.001	0.79 (−0.09–1.67)	0.57 (−0.29–1.44)	0.194	0.05 (0.02–0.08)	0.02 (−0.01–0.06)	0.058
T3	0.40 (0.17–0.64)	0.25 (0.05–0.46)	0.017	1.30 (0.38–2.22)	1.14 (0.24–2.05)	0.013	0.04 (0.01–0.07)	0.01 (−0.01–0.04)	0.298
uPDI									
T1	ref	ref		ref	ref		ref	ref	
T2	−0.12 (−0.35–0.11)	−0.12 (−0.32–0.08)	0.233	−0.57 (−1.46–0.32)	−0.48 (−1.35–0.39)	0.283	−0.01 (−0.03–0.02)	−0.00 (−0.03–0.02)	0.815
T3	−0.43 (−0.67–−0.19)	−0.27 (−0.48–−0.07)	0.009	−1.05 (−1.98–−0.13)	−0.80 (−1.71–0.11)	0.084	−0.04 (−0.06–−0.01)	−0.01 (−0.04–0.01)	0.144

T: tertile; PDI: plant-based diet index; hPDI: healthy PDI; uPDI: unhealthy PDI; SPPB: Short Physical Performance Battery. Model-1 was adjusted for age, sex, race, and education; Model-2 included further adjustments for age, sex, race, education, smoking status, physical activity, alcohol intake, number of chronic diseases, BMI, years since the first dietary assessment, and average energy intake. * *p*-value for Model-2.

## Data Availability

The dataset is publicly available through controlled access by request, pending approval by the BLSA cohort investigators and the NIA research team via https://www.blsa.nih.gov/.

## Supplementary Materials

The following supporting information can be downloaded at https://www.mdpi.com/article/10.3390/nu16234249/s1: Supplementary Table S1: Overview of food items constituting the 18 food groups; Supplementary Table S2: Food group intake in portion sizes according to plant-based diet indices.
