# Supplementary material for: Plant-Based Diets and Their Associations with Physical Performance in the Baltimore Longitudinal Study of Aging
Source: Nutrients. 2024 Dec 9;16(23):4249. doi: 10.3390/nu16234249 (PMC11644655; doi:10.3390/nu16234249)
Supplement: Supplementary file 1 [file nutrients-16-04249-s001.zip › nutrients-3343245-supplementary.pdf]

Plant-Based Diets and Their Associations with Physical Performance in the Baltimore Longitudinal Study of Aging

Galya Bigman [1,*], Marius Emil Rusu [2], Amber S. Kleckner [3], John D. Sorkin [1,4,5], Yichen Jin [6], Sameera A. Talegawkar [6], Toshiko Tanaka [7], Luigi Ferrucci [7] and Alice S. Ryan [4,5]

Supplementary Table S1. Overview of food items constituting the 18 food groups.

	Food Groups	Overall Plant-Based Diet Index (PDI)	Healthy Plant-Based Diet Index (hPDI)	Less Healthy Plant-Based Diet Index (uPDI)
Healthy Plant Food Groups	Whole Grains	Positive	Positive	Reverse
	Fruits	Positive	Positive	Reverse
	Vegetables	Positive	Positive	Reverse
	Nuts	Positive	Positive	Reverse
	Legumes	Positive	Positive	Reverse
	Vegetable Oil			
	Tea and Coffee	Positive	Positive	Reverse
Less Healthy Plant Food Groups	Fruit Juices	Positive	Reverse	Positive
	Sugar-Sweetened and Artificially Sweetened Beverages	Positive	Reverse	Positive
	Refined Grains	Positive	Reverse	Positive
	Potatoes	Positive	Reverse	Positive
	Sweets and Desserts	Positive	Reverse	Positive
Animal Food Groups	Animal Fat	Reverse	Reverse	Reverse
	Dairy	Reverse	Reverse	Reverse
	Eggs	Reverse	Reverse	Reverse
	Fish or Seafood	Reverse	Reverse	Reverse
	Meat (Poultry and Red Meat)	Reverse	Reverse	Reverse
	Miscellaneous Animal Foods	Reverse	Reverse	Reverse

Supplementary Table S2. Food Group intake in average servings according to plant-based diet indices.

Plant Food Groups	PDI			p-value	hPDI			p-value	uPDI			p-value
						Mean (SD)						
**Healthy**	**T1**	**T2**	**T3**		**T1**	**T2**	**T3**		**T1**	**T2**	**T3**	
**Whole Grain**	1.65	1.71	2.08	<0.001	1.36	1.86	2.22	<0.001	2.00	1.77	1.63	<0.001
	(1.13)	(1.12)	(1.19)		(0.82)	(1.19)	(1.28)		(1.13)	(1.02)	(1.29)	
**Fruit**	0.98	1.26	1.68	<0.001	0.76	1.23	1.90	<0.001	1.86	1.17	0.78	<0.001
	(0.91)	(0.92)	(1.07)		(0.57)	(0.94)	(1.10)		(1.04)	(0.92)	(0.71)	
**Vegetables**	2.53	2.73	3.20	<0.001	1.97	2.71	3.79	<0.001	3.80	2.56	1.99	<0.001
	(1.42)	(1.58)	(1.60)		(0.98)	(1.33)	(1.71)		(1.61)	(1.26)	(1.13)	
**Nuts**	0.55	0.64	0.87	<0.001	0.36	0.64	1.05	<0.001	0.96	0.56	0.48	<0.001
	(0.68)	(0.61)	(0.82)		(0.37)	(0.63)	(0.91)		(0.85)	(0.58)	(0.59)	
**Bean**	0.14	0.19	0.25	<0.001	0.11	0.17	0.30	<0.001	0.25	0.17	0.14	<0.001
	(0.13)	(0.19)	(0.27)		(0.10)	(0.15)	(0.28)		(0.25)	(0.17)	(0.15)	
**Vegetable Oil**	0.18	0.24	0.35	<0.001	0.16	0.23	0.37	<0.001	0.33	0.23	0.18	<0.001
	(0.21)	(0.32)	(0.39)		(0.19)	(0.28)	(0.40)		(0.28)	(0.26)	(0.37)	
**Tea and Coffee**	1.70	1.45	1.94	<0.001	1.33	1.60	2.20	<0.001	2.21	1.54	1.33	<0.001
	(1.82)	(1.34)	(1.91)		(1.37)	(1.53)	(2.11)		(1.99)	(1.48)	(1.53)	
**Less healthy**												
**Juices**	0.42	0.51	0.65	<0.001	0.54	0.52	0.48	0.004	0.36	0.52	0.67	<0.001
	(0.83)	(0.68)	(0.89)		(0.77)	(0.74)	(0.92)		(0.59)	(0.72)	(1.03)	
**Refined Grain**	4.56	4.27	4.32	<0.001	4.56	4.37	4.24	<0.001	3.87	4.13	5.20	<0.001
	(2.31)	(2.51)	(2.28)		(2.23)	(2.45)	(2.40)		(1.97)	(2.11)	(2.74)	
**Potatoes**	0.33	0.31	0.30	<0.001	0.38	0.32	0.25	<0.001	0.23	0.31	0.42	<0.001
	(0.28)	(0.29)	(0.28)		(0.30)	(0.28)	(0.25)		(0.20)	(0.24)	(0.35)	
**Sugar Sweetened**	0.73	0.75	0.91	<0.001	0.96	0.79	0.60	<0.001	0.55	0.69	1.13	<0.001
**Beverages**	(1.33)	(1.21)	(1.26)		(1.29)	(1.28)	(1.22)		(1.05)	(0.99)	(1.62)	
**Sweets and Des-**	0.88	0.89	0.99	<0.001	1.02	0.93	0.78	<0.001	0.70	0.83	1.22	<0.001
**serts**	(0.83)	(0.72)	(0.84)		(0.84)	(0.81)	(0.73)		(0.60)	(0.68)	(0.98)	
**Animal Food Groups**												
**Animal Fat**	0.08	0.07	0.06	<0.001	0.08	0.07	0.06	<0.001	0.09	0.06	0.06	<0.001
	(0.10)	(0.08)	(0.08)		(0.09)	(0.08)	(0.10)		(0.10)	(0.07)	(0.08)	
**Dairy**	2.37	1.92	1.87	<0.001	1.96	2.10	2.21	<0.001	2.36	2.02	1.87	<0.001
	(1.34)	(1.11)	(1.12)		(1.14)	(1.19)	(1.35)		(1.19)	(1.15)	(1.30)	
**Egg**	0.47	0.33	0.22	<0.001	0.34	0.38	0.34	0.018	0.40	0.33	0.33	<0.001
	(0.47)	(0.29)	(0.20)		(0.32)	(0.40)	(0.40)		(0.30)	(0.38)	(0.42)	
**Fish and Seafood**	0.43	0.33	0.27	<0.001	0.34	0.38	0.34	0.004	0.41	0.33	0.31	<0.001
	(0.37)	(0.22)	(0.23)		(0.21)	(0.39)	(0.28)		(0.29)	(0.23)	(0.35)	
**Meat**	1.32	0.94	0.76	<0.001	1.21	1.00	0.87	<0.001	1.04	0.97	1.10	<0.001
	(0.76)	(0.58)	(0.52)		(0.72)	(0.67)	(0.61)		(0.67)	(0.64)	(0.74)	
**Miscellaneous**				<0.001				<0.001				<0.001
**Animal-Based**	0.62	0.48	0.38		0.53	0.51	0.46		0.64	0.46	0.41	
**Foods**	(0.50)	(0.44)	(0.39)		(0.44)	(0.48)	(0.46)		(0.51)	(0.42)	(0.42)	

T: Tertile; PDI: Plant-Based Diet Index; hPDI: Healthy Plant-Based Diet Index; uPDI: Unhealthy Plant-Based Diet Index; SPPB: Short Physical Performance Battery.
